# Modeling and Parameter Analysis of Basic Single Channel Neuron Mass Model for SSVEP

**DOI:** 10.3390/s25061706

**Published:** 2025-03-10

**Authors:** Depeng Gao, Yujuan Wang, Peirong Fu, Jianlin Qiu, Hongqi Li

**Affiliations:** 1 School of Yonyou Digital and Intelligence, Nantong Institute of Technology, Nantong 226000, China; gaodepeng@ntit.edu.cn (D.G.); qiujl@ntit.edu.cn (J.Q.); 2 School of Software, Northwestern Polytechnical University, Xi’an 710000, China; wangyujuan12@mail.nwpu.edu.cn (Y.W.); 18406430685@163.com (P.F.)

**Keywords:** steady-state visually evoked potential, neural mass model, model parameters, parameter analysis, motion control

## Abstract

While steady-state visual evoked potentials (SSVEPs) are widely used in brain–computer interfaces (BCIs) due to their robustness to rhythmic visual stimuli, their generation mechanisms remain poorly understood. Challenges such as experimental complexity, inter-subject variability, and limited physiological interpretability hinder the development of efficient BCI systems. This study employed a single-channel neural mass model (NMM) of V1 cortical dynamics to investigate the biophysical underpinnings of SSVEP generation. By systematically varying synaptic gain, time constants, and external input parameters, we simulated *δ/α/γ* band oscillations and analyzed their generation principles. The model demonstrates that synaptic gain controls oscillation amplitude and harmonic content, and time constants determine signal decay kinetics and frequency precision, while input variance modulates harmonic stability. Our results reveal how V1 circuitry generates frequency-locked SSVEP responses through excitatory–inhibitory interactions and dynamic filtering mechanisms. This computational framework successfully reproduces fundamental SSVEP characteristics without requiring multi-subject experimental data, offering new insights into the physiological basis of SSVEP-based brain–computer interfaces.

## 1. Introduction

The brain is an extremely complex organ containing billions of interconnected neurons. To understand and analyze brain activity, one can abstract and simplify the brain through mathematical models by accurately expressing the interactions and information transfer between neurons. Theoretically, different brain electrical activities can be simulated by adjusting the parameters of the model to mimic the brain.

The neuronal models widely used to model electroencephalograph (EEG) activity can be divided into two main categories. The first one focuses on the micro level, including Hodgkin–Huxley [1], Chay [2], Traub [3], and so on, which can be used to understand the physiological mechanisms of the brain from the perspective of neurons. However, the brain is a highly complex system comprising hundreds of billions of neurons and hundreds of trillions of synapses. These neurons connect to form a vast network; therefore, it is very difficult to capture such a complex network structure and dynamic characteristics when modeling at the neuron level.

The second model type treats a mass of neurons in the brain as a single unit whose activities are described by a set of aggregated parameters, i.e., the lumped parameter model. The neural mass model (NMM) is based on neural populations and uses the lumped state variable as the average approximate behavior of a specific neuronal mass [4,5]. Rather than modeling individual microscopic neurons, it captures the overall characteristics of the neural mass composed of specific cell types. The NMM also accounts for interactions between different neural masses, enabling the simulation of large-scale neural network dynamics at a macro level. Wilson and Cowan explored the excitatory and inhibitory interactions within local neuronal populations, deriving coupled nonlinear differential equations that describe these interactions through excitatory and inhibitory neuronal models [6]. Silva et al. introduced a population-based neuronal structure for simulating *α* rhythmic EEG signals consisting of interconnected excitatory and inhibitory neuronal populations, where each subpopulation is modeled by a first-order differential equation containing a static nonlinear function [7,8]. This model is physiologically grounded and is widely used as a basic single-channel neuron mass model. Jansen et al. further refined the NMM, demonstrating that a mathematical model of neuronal mass could effectively simulate EEG activity and generate visual evoked potential (VEP) waveforms. Moreover, they also found that the ability to simulate VEP activity was primarily related to excitatory connections within the cortex [9].

Steady-state visual evoked potentials (SSVEPs) refer to the EEG electrical signals evoked by the visual stimuli of specific frequencies. When the retina receives visual stimuli ranging from 3.5 Hz to 75 Hz, the brain generates electrical activity at the same frequency or harmonics. This neural response arises from the synchronization of cortical neurons in the primary visual cortex (V1), where periodic visual inputs drive *γ*-band oscillations (30–80 Hz) through thalamocortical pathways [10]. The resulting SSVEP amplitude is modulated by attentional mechanisms as subjects maintain a focused gaze on the stimuli [11].

Brain–computer interfaces (BCIs) based on SSVEP achieve high information transfer rates by monitoring subjects’ responses to light stimuli at multiple frequencies. The high signal-to-noise ratio (SNR) of SSVEP signals in the frequency domain enables robust detection of user intent, significantly improving BCI accuracy. These systems typically do not require complex cognitive tasks by the subjects, only the maintenance of attention to visual stimuli at specific frequencies. This attentional focus enhances neural entrainment in the visual cortex, further amplifying the SSVEP response through top-down modulation from the parietal and frontal regions [12]. Hence, the fatigue and cognitive burden of the subjects are minimized. Compared to other EEG signals, SSVEPs are relatively stable and reliable, and their system response speed is fast, making them suitable for scenarios requiring rapid feedback and real-time applications. For instance, the ability to encode multiple commands via distinct stimulus frequencies (e.g., 10 Hz, 12 Hz, 15 Hz) allows SSVEP-BCIs to achieve high-dimensional control without prolonged user training. This enhances the stability and availability of BCI systems. Consequently, SSVEPs have been widely used in the development of various brain-controlled intelligent devices [13], such as brain-controlled robotic arms [14], brain-controlled quadcopters [15], brain-controlled wall-climbing robots [16], brain-controlled prosthetics [17], brain-controlled wheelchairs [18], brain-controlled smart homes [19], and other systems [20,21].

However, despite the great potential of SSVEP-BCIs, several challenges remain, including experimental difficulties and the limited availability of subjects. Firstly, the experimental difficulties refer to the need to carry out complex experimental setups and extensive signal processing. In the SSVEP-BCI, subjects are often required to look at visual stimuli with different frequencies. Accurately presenting the stimuli and acquiring the signals during this process requires designing and executing the designated experiments within a highly controllable laboratory environment. This complexity may lead to technical and operational challenges in the experimental process, further affecting the advancement of research. Secondly, recruiting sufficient and diverse subjects is essential to validating and promoting the SSVEP-BCI technology in practical applications. However, the recruitment of subjects is limited by many economic factors such as time, money, and manpower. The types of subjects are also limited by age, sex, and health. This restricts the breadth and reliability of SSVEP-BCI research.

The study of a neural mass model by Jansen et al. [9] found that the NMM can be used to describe the interaction between neuron populations and is suitable for explaining neural activity in the cerebral cortex. In addition, by simulating the collective behaviors of neural populations, the NMM can pick up oscillations with specific frequencies produced in response to visual stimuli. This understanding of the neural mass provides an effective tool for more accurately understanding and modeling the neural activity of the brain when perceiving steady-state visual evoked potentials. The main contributions of this paper are as follows:

(1) We explore the mechanism for generating SSVEPs based on the NMM, which helps to construct the SSVEP by artificially generating virtual signals similar to actual EEGs. As a result, it does not need an experimental setup, subject recruitment, and equipment configuration, and the simulation modeling method provides a more cost-effective avenue for SSVEP-BCI research, which will improve repeatability.
(2) The principle of the neural mass model for single-channel SSVEP is analyzed in detail, where the three basic dynamic waveforms are generated by the model, and the effects of key parameters are analyzed on the simulated signals.
(3) We provide current study limitations and further research directions, which help to clarify the understanding of the physiological significance and functionality of the current NMM.

The remainder of the paper is organized as follows: Section 2 provides the basic neural mass model employed in the construction of SSVEP and a detailed illustration of the structure of the model. The specific simulation results are provided in Section 3, complete with a thorough analysis of each key parameter’s influence. Section 4 provides the conclusion to the study, including a discussion of the limitations of the current study and future research directions.

## 2. Neural Mass Model

Human functions such as thinking, perception, emotion, memory, and movement are mediated by electrical signal transmission and interaction between neurons. A neuron is the basic constituent unit of the neural system, typically comprising structures such as the cell body, dendrites, and axon. These components work in concert to receive, integrate, and transmit neural signals. Neurons communicate with each other efficiently and complexly via specialized connection structures known as synapses. Within the neural system, a neural mass is a functional grouping of neurons connected through synaptic connections. The neural masses usually work together to perform a specific neural activity or process specific information. There are two main types of cells in a neural mass: pyramidal cells and non-pyramidal cells. Pyramidal cells’ shapes are usually triangular or conical. Their characteristic synaptic morphology allows them to play an important role in transmission and long-range connection. Pyramidal cells predominantly release excitatory neurotransmitters, which activate downstream neurons. Non-pyramidal cells focus more on local neural modulation, which includes many types of neurons. Some of them are excitatory, releasing excitatory neurotransmitters that activate neighboring neurons, thereby enhancing signal transmission, while others are inhibitory, releasing inhibitory neurotransmitters that suppress the activity of neighboring neurons. These inhibitory cells help regulate and maintain the balance and stability of the neural network. The interactions between excitatory and inhibitory processes form the basis of information processing within neuron masses. Through the collaborative work of both cell types, the neural system can achieve complex and precise signal transmission, thereby enabling the execution of different brain functions [6].

### 2.1. Single-Channel Basic Neuron Mass Model

**Figure 1:**
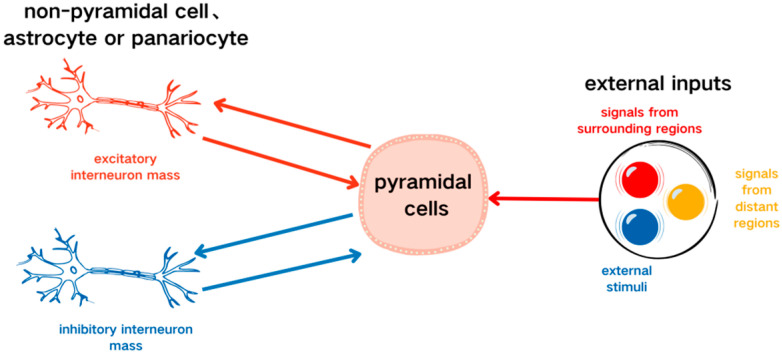
Single-channel basic neuron mass feedback model.

In the single-channel basic neuron mass model described by Jansen et al. [9], the pyramidal cell subpopulation receives excitatory and inhibitory inputs from interneuron subpopulations within the same region. Simultaneously, the outputs of this pyramidal cell subpopulation provide feedback to the excitatory and inhibitory interneuron subpopulations. The excitatory interneuron subpopulations also receive input from other regions or external stimuli, as shown in Figure 1.

**Figure 2:**
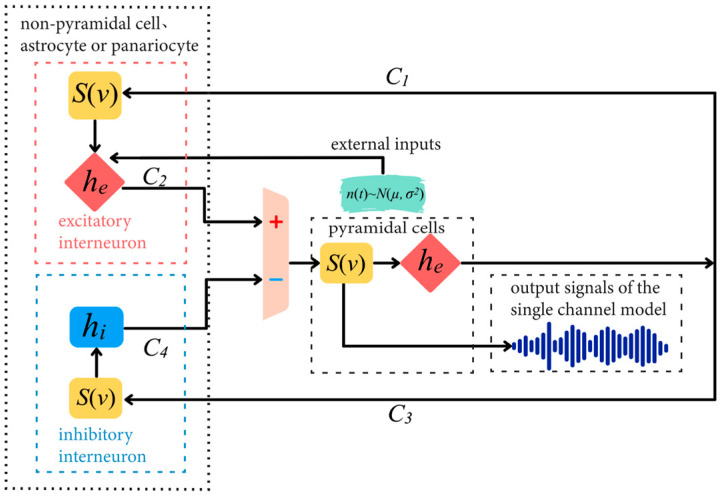
Block diagram of a single-channel basic neuron mass.

In the single-channel basic neuron mass model, Gaussian white noise *n(t)∼N(μ, σ^2^)* is used as the excitatory input to represent all external stimuli and signals in the uncertain region, while the inhibitory connections between these signals are ignored when modeling. As shown in Figure 2, *C*_1_ and *C*_2_ represent the connectivity coefficients between the pyramidal cell subpopulation and excitatory interneuron subpopulation, reflecting the proportional relationship between the average numbers of synapses in the two subpopulations. The connection between the pyramidal cell subpopulation and the inhibitory interneuron subpopulation is represented by two additional connectivity coefficients, *C*_3_ and *C*_4_, which correspondingly reflect the proportional relationship between the pyramidal cell subpopulation and the excitatory interneuron subpopulation [22].

As shown in Figure 2, single-channel basic neuron mass modeling includes a feedback mechanism, physiological parameters, and two information transformation processes. The concrete modeling method is as follows:

(1) Static nonlinear function. The average membrane voltage of the neuron mass is converted into the average pulse density of the action potential. This information transformation is represented by the following instantaneous S-function:

(1) $$S(v)=\frac{2e_{0}}{1+e^{r\left(v_{0}-v\right)}}$$

where *S(v)* is a static nonlinear function representing the average pulse density of the action potential, *v* represents the pre-synaptic average membrane voltage, *e*_0_ represents the maximum firing rate of the neuron mass, *e* is the base value of the natural logarithm function, *r* represents the curvature of the S-function, and *v*_0_ is the postsynaptic membrane voltage corresponding to the firing rate *e*_0_.

**Figure 3:**
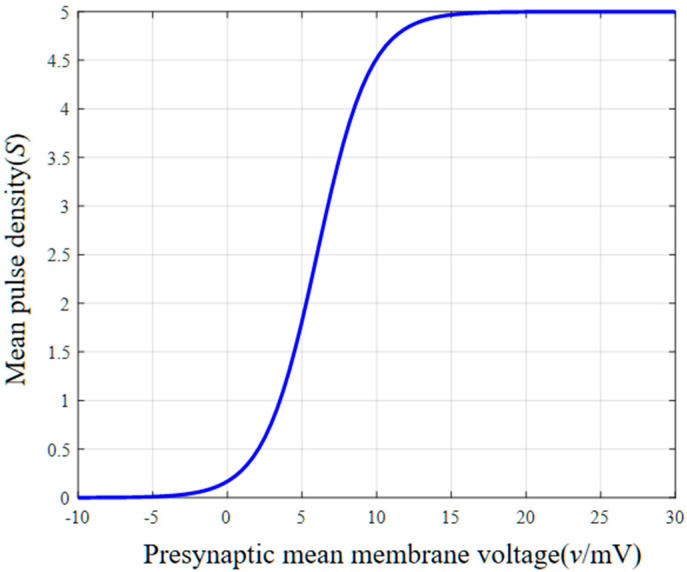
Static nonlinear function.

Figure 3 illustrates the trend of the mean pulse density as a function of the pre-synaptic average membrane voltage. When the pre-synaptic mean membrane voltage *v* < −5 mV, the mean pulse density *S(v)* = 0. When *v* > −5 mV, the mean pulse density *S*(*v*) increases gradually with the increase in the pre-synaptic mean membrane voltage. The growth rate increases and then decreases, reaching its maximum between 5 mV and 10 mV. Beyond 10 mV, the growth rate gradually slows down until the maximum average pulse density is reached, i.e., 2*e*_0_ = 5, and then the static nonlinearity reaches the saturation state, and the average pulse density no longer changes with the increase in the pre-synaptic average membrane voltage. There is a nonlinear mapping relationship between the mean pulse density and the mean pre-synaptic membrane voltage; the S-function presents a nonlinear feature, which can verify the nonlinearity of the EEG signal.

(2) Dynamic linear function. The average pulse density of the pre-synaptic membrane is converted into excitatory or inhibitory postsynaptic membrane voltage, which occurs at the synapses of neurons. Equations (2) and (3) show the impulse responses of the linear function to excitability and inhibition, respectively.

(2) $$h_{e}(t)=u(t)\eta_{e}te^{-t\omega_{e}}$$

(3) $$h_{i}(t)=u(t)\eta_{i}te^{-t\omega_{i}}$$

In Equation (2), *h_e_(t)* represents the excitatory postsynaptic membrane impulse response; *u(t)* represents the Heaviside function, which is used to describe the mutation or step change in the system behavior; *ω_e_* represents the reciprocal of the mean time constant, which is used to describe the influence of the mean time constant of the excitatory membrane and the mean distribution delay of dendrites; and *η_e_* is the product of the excitatory mean synaptic gain *G_e_* and *ω_e_*.

In Equation (3), *h_i_*(*t*) represents the inhibitory postsynaptic membrane impulse response; *ω_i_* represents the reciprocal of the mean time constant, which is used to describe the influence of the mean time constant of the inhibitory membrane and the mean distribution delay of dendrites; and *η_i_* is the product of the inhibitory mean synaptic gain *G_i_* and *ω_i_*.

In the following section, we will investigate the effects of the average synaptic gain *G_e_* and the reciprocal of the average time constant *ω_e_* on the dynamic linear function.

**Figure 4:**
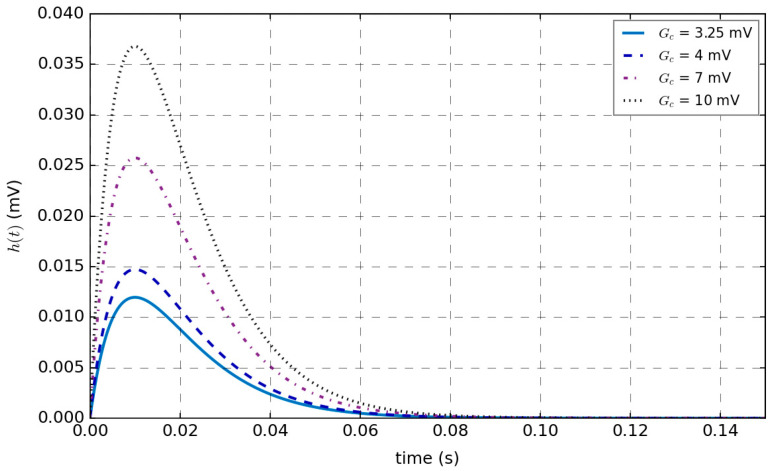
Changes in the impulse response of the linear function with average synaptic gain *G_e_*.

The variation curve of *h* (i.e., the impulse response of the linear function) with average synaptic gain *G_e_* is shown in Figure 4, where the average time constant *ω_e_* = 0.01 s^−1^, and the values of *G_e_* are set to 3.25 mV, 4 mV, 7 mV, and 10 mV. For the same *G_e_*, the value of *h* increases with time. *h* reaches its maximum value when the time is *ω_e_* and then gradually decreases to zero. As the average synaptic gain *G_e_* increases, the peak value of the impulse response function *h* also increases during the same time *t*; that is, the maximum value of the postsynaptic membrane voltage increases.

**Figure 5:**
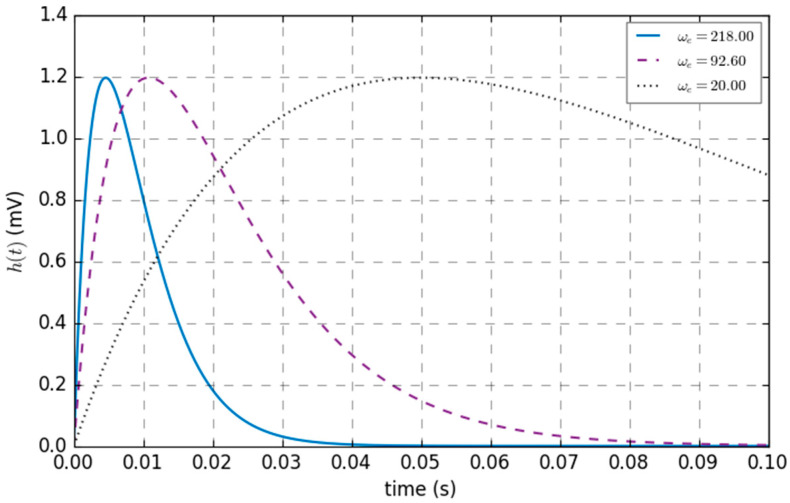
Changes in the impulse response of the linear function with the average time constant *ω_e_*.

The variation curve of *h* with the average time constant *ω_e_* is shown in Figure 5, where average synaptic gain *G_e_* = 3.25 mV, and the values of *ω_e_* are set to 20.00, 92.60, and 218.00 s^−1^. For the same *ω_e_*, the amplitude increases with time until *ω_e_*, at which point *h* reaches its maximum value. After that, *h* gradually decreases with time until it reaches zero. If the linear function has different *ω_e_*^−1^ values, the time to reach the maximum value will change accordingly. With the increase in *ω_e_*, the times will shift backward, and the linear function will reach the maximum value at *ω_e_*. Thus, adjusting *ω_e_* can regulate the sensitivity of the synaptic gain, and the delay time can be adjusted by this linear function.

The dynamic linear transformation function *h*(*t*) can be further represented by a first-order ordinary differential equation, as shown in Equation (4).

(4) $$\left\{\begin{array}{l} \bar{z}(t)=z_{1}(t)\\ {\bar{z}}_{1}(t)=\eta x(t)-2\omega z_{1}(t)-\omega^{2}z(t) \end{array}\right.$$

where *x*(*t*) and *z*(*t*) are the input and output signals of the dynamic linear function, respectively, *z*_1_(*t*) is the first order derivative of *z*(*t*), *ω* represents the reciprocal of the mean time constant, *η* represents the product of the mean synaptic gain *G_e_* and *ω*, and the excitability or inhibition of *η* and *ω* is determined by the properties of the interneuron. Equation (4) describes the potential conversion completed on the synapses, through which the input and output of the linear transformation process can be more intuitively understood, and the information transformation process of the dynamic linear transformation can be more clearly represented.

The input x(t) in Equation (4) varies across subpopulations, as shown in Figure 2. For excitatory interneurons, the input $x_{excitatory}(t)$ integrates two critical components that drive the model’s dynamics. First, the external stimulus n(t) is modeled as Gaussian white noise. Second, feedback signals from pyramidal cells are incorporated through the term $C_{1}S(y_{0}(t))$, where $y_{0}(t)$ represents the output potential of pyramidal cells.

(5) $$x_{excitatory}(t)=n(t)+C_{2}S(C_{1}y_{0}(t))$$

The resultant postsynaptic potential $y_{1}(t)$ reflects the excitatory subpopulation’s contribution to the network’s oscillatory behavior, directly influencing the generation of SSVEP’s fundamental frequency.

(6) $$\left\{\begin{array}{l} {\bar{y}}_{1}(t)=y_{4}(t)\\ {\bar{y}}_{4}(t)=\eta_{e}x_{excitatory}(t)-2\omega_{e}y_{4}(t)-{\omega_{e}}^{2}y_{1}(t) \end{array}\right.$$

The input $x_{inhibitory}(t)$ in the inhibitory interneuron subpopulation is exclusively derived from feedback signals originating in pyramidal cells.

(7) $$x_{inhibitory}(t)=C_{4}S\left(C_{3}y_{0}(t)\right)$$

This input is processed through the dynamic linear response (Equation (4)), generating the inhibitory postsynaptic potential $y_{2}(t)$, governed by the following system of equations:

(8) $$\left\{\begin{array}{l} {\bar{y}}_{2}(t)=y_{5}(t)\\ {\bar{y}}_{5}(t)=\eta_{i}x_{inhibitory}(t)-2\omega_{i}y_{5}(t)-{\omega_{i}}^{2}y_{2}(t) \end{array}\right.$$

Pyramidal cells receive input $x_{pyramidal}(t)$ from the difference between the postsynaptic excitatory and inhibitory subpopulations.

(9) $$x_{pyramidal}(t)=S(y_{1}(t)-y_{2}(t))$$

Here, $y_{1}(t)-y_{2}(t)$ reflects the dynamic balance between excitation and inhibition, which is the output of the NMM. The dynamics of pyramidal cells are described by the following:

(10) $$\left\{\begin{array}{l} {\bar{y}}_{0}(t)=y_{3}(t)\\ {\bar{y}}_{3}(t)=\eta_{e}x_{pyramidal}(t)-2\omega_{e}y_{3}(t)-{\omega_{e}}^{2}y_{0}(t) \end{array}\right.$$

In Equations (5)–(10), *y*_3_(*t*), *y*_4_(*t*), and *y*_5_(*t*) are first derivatives of *y*_0_(*t*), *y*_1_(*t*), and *y*_2_(*t*), respectively; *ω_e_* is the reciprocal of the average time constant, which is used to describe the effect of the mean time constant of the excitatory cell membrane and the mean distribution delay of dendrites; *η_e_* = *G_e_* * *ω_e_* is the product of the excitatory mean synaptic gain and the reciprocal of the mean time constant; *ω_i_* is the reciprocal of the average time constant, which is used to describe the effect of the mean time constant of the inhibitory cell membrane and the mean distribution delay of the dendrites; *η_i_* = *G_i_* * *ω_i_* is the product of the inhibitory mean synaptic gain and the reciprocal of the mean time constant; *n*(*t*) represents signals and external stimuli from all variable regions; and *C*_1_, *C*_2_, *C*_3_, and *C*_4_ represent the connectivity coefficients between the pyramidal cell subpopulation and the interneuron subpopulation.

### 2.2. Parameters of a Single-Channel Basic Neuron Mass Model

To explore the effect of variable model parameters on outputs, it is necessary to identify which parameters in the single-channel basic neuron mass model are related and which parameters have standard values.

The relationships among *C*_1_–*C*_4_ have been investigated in several studies. First, *C*_1_ and *C*_3_ represent the number of synapses on the dendrites from the pyramidal cell mass to the excitatory and inhibitory interneuron masses, respectively. *C*_2_ and *C*_4_ are positively correlated with the number of synapses on the dendrites from the excitatory and inhibitory interneuron masses to the pyramidal cell mass, respectively. Typically, *C*_2_ includes the synapses of a thalamic origin, where the researcher generally indicates the count as *C*_2_’.

Braitenberg and Schüz studied the pyramidal cells in the visual cortex of mice and found that *C*_1_ + *C*_3_ = *C*_2_ + *C*_2_’ + *C*_4_ [23]. White observed that synapses produced in the spine are located in excitatory cells; however, synapses produced in the axis may also be located in inhibitory cells [24]. Elhanany and White found that in the somatic motor cortex of mice, 87% of the synapses are in the spine, and the remaining 13% are in the axis [25], indicating that about 6.5% of the synapses formed by pyramidal cells are inhibitory; thus, we have *C*_3_/(*C*_1_ + *C*_3_) = 6.5/100. Later, Liu et al. found that 80% of synapses on the dendrites of pyramidal cells in cats’ motor cortex are excitatory [26]; hence, (*C*_2_ + *C*_2_’)/[(*C*_2_ + *C*_2_’) + *C*_4_] = 0.8.

Larkman’s study showed that most excitatory cells in the visual cortex are pyramidal [27]; that is, most cells in the excitatory feedback circuit are pyramidal. If the cells in the circuit are consistent with synapses, the pyramidal cells have the same number of synapses as other cells (such as non-pyramidal cells, astrocytes, or panariocytes) in the circuit. The number of synapses from the pyramidal cell to the excitatory interneuron mass should be the same as the number of synapses from the excitatory interneuron mass to the pyramidal cell, i.e., *C*_1_ = *C*_2_ + *C*_2_’.

According to [28,29], about 20% of the asymmetric synapses in the fourth layer of the cortex are generated by thalamocortical terminals; thus, *C*_2_’/(*C*_2_ + *C*_2_’) = 0.2, i.e., *C*_2_’ = *C*_2_/4.

The transformation of the above formulas gives us *C*_2_/*C*_1_ = 0.8 and *C*_3_ = *C*_4_. However, heterogeneity in experimental subjects (e.g., age, neurological status), inconsistent criteria for pyramidal cell selection (morphological vs. electrophysiological markers), and diversity in imaging techniques and analytical pipelines collectively contribute to synaptic count variability, complicating the establishment of universal synaptic scaling relationships in excitatory and inhibitory feedback loops. For example, we can obtain *C*_1_ = 14.4*C*_3_ because *C*_3_/(*C*_1_ + *C*_3_) = 6.5/100; however, taking *C*_1_ = *C*_2_ + *C*_2_’ and *C*_3_ = *C*_4_ into (*C*_2_ + *C*_2_’)/[(*C*_2_ + *C*_2_’) + *C*_4_] = 0.8 will give *C*_1_ = 4*C*_3_. Given these differences, *C*_1_ = 4*C*_3_ is selected here, where we assume that the relationship is more suitable for the human user when producing the interested signals; hence, we get *C*_1_ = *C*_2_/0.8 = 4*C*_3_ = 4*C*_4_.

Indeed, the functions of the synapses are affected by many factors, such as number, structure, and characteristics. Changing variable *C* can affect these factors, further affecting the occurrence and evolution of synaptic phenomena. The flexibility of variable *C* enables it to adapt to diverse physiological environments and produce significant effects under different conditions, especially in regulating synaptic function. Here, when *C* = *C*_1_, then *C*_2_ = 0.8*C*, *C*_3_ = 0.25*C*, and *C*_4_ = 0.25*C*.

From the dynamic linear transformation function, we can see that the amplitude of the impulse response of the linear function is proportional to the excitatory average synaptic gain G*_e_* and the inhibitory average synaptic gain *G_i_*. Rotterdam et al. proposed *G_e_* = 3.25 mV and *G_i_* = 22 mV [30]; however, Dodt et al. found that some neuropeptides may change the amplitude of the postsynaptic membrane voltage [31], and thus *G_e_* and *G_i_* cannot be strictly limited. Since *ω_e_* and *ω_i_* represent the reciprocals of the excitability mean time constant and the inhibition mean time constant, respectively, *ω_e_* and *ω_i_* are inversely proportional to the time required for the postsynaptic membrane voltage to reach its maximum value. Because the average synaptic gain and the average time constant do not change easily over short periods, they can be set to fixed values [32]; therefore, *ω_e_* = 100 s^−1^ and *ω_i_* = 50 s^−^^1^ in this study.

Many substances can affect the excitability of neurons [33,34]; for example, the change in *v*_0_ in the nonlinear function will affect the ignition threshold. Thus, it is necessary to determine the parameters involved in the nonlinear function. In most cases, *v*_0_ = 6 mV, *e*_0_ = 2.5 s^−1^, and *r* = 0.56 mV^−1^, which we adopted in our current study.

## 3. Results

According to the single-channel basic neuron mass model, the EEG signals with δ, α, and γ rhythms can be output by adjusting different physiological parameters in the model. To simulate the single-channel basic neuron mass model in this study, the Runge–Kutta solver in MATLAB/Simulink is used, and the basic sampling time is set to 1/1024 s. The external input is represented by Gaussian white noise *n*(*t*)~*N*(*μ*,*σ*^2^), where *μ* = 220 and *σ*^2^ = 100 [22]. The standard physiological parameters of the EEG are taken as the model parameters and are set to the widely used values in the basic neuron mass model. The model parameters and their values are shown in Table 1.

**Figure 6:**
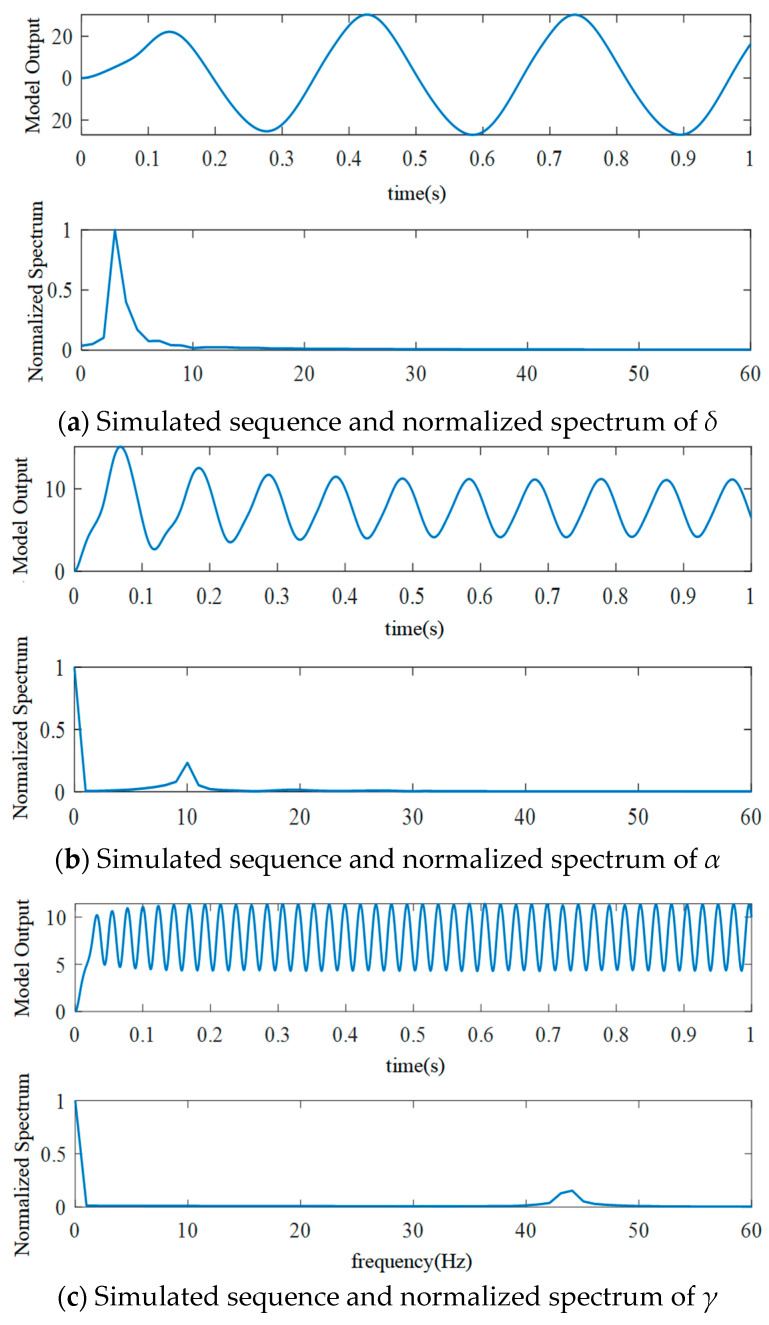
Simulated sequences and the normalized spectrum of *δ*, *α*, and *γ*.

By adjusting *η_e_*, *η_i_*, *ω_e_*, and *ω_i_* of the dynamic linear transformation function, the basic single-channel neuron mass model can output various types of simulated EEG signals with δ, α, or γ rhythms. When *η_e_*, *η_i_*, *ω_e_*, *ω_i_*, and external input *n*(*t*) are set to the typical values provided in Table 1, the neuron mass model outputs three basic dynamic waveforms that are significantly different in the time domain and frequency domain (Figure 6). In the following section, we will analyze the effect of each parameter on the model in detail.

### 3.1. The Effect of η_e_ on the Model

**Figure 7:**
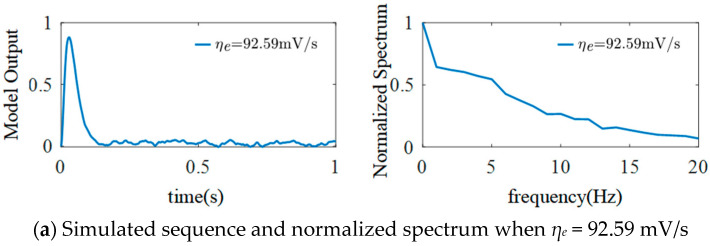

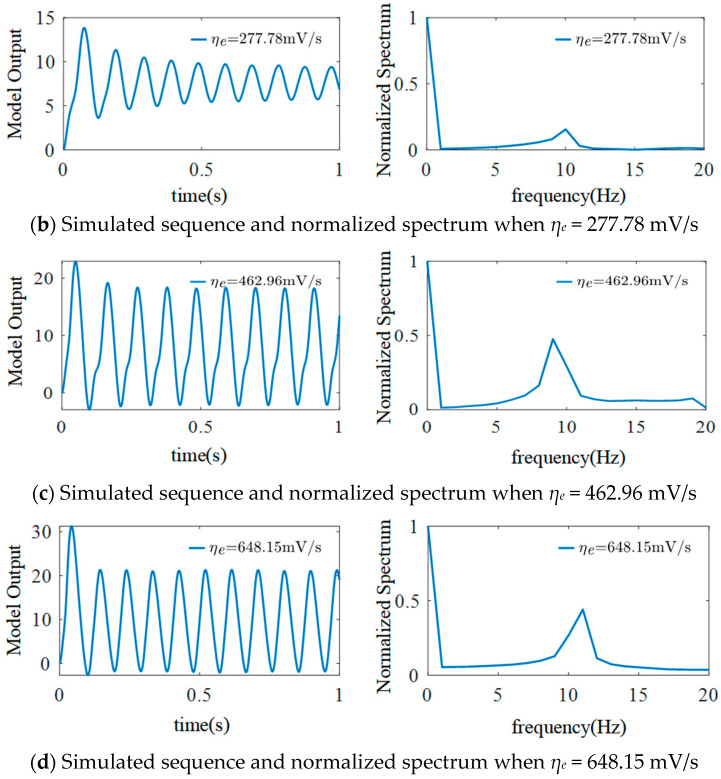
Simulated EEG signal curves and the normalized spectrum with different *η_e_*.

For the simulated EEG signals, *μ* = 220, *σ*^2^ = 100, *η_i_* = 1100 mV/s, *ω_e_* = 100 s^−1^, *ω_i_* = 50 s^−1^, the basic sampling time is adjusted to 1/1024 s, and other parameters are selected as shown in Table 1. To explore the influence of *η_e_* on the dynamic behavior of the model, the value of the excitatory synaptic gain *G_e_* is adjusted by changing the value of *η_e_*. The simulation results are shown in Figure 7.

Based on Figure 7, different EEG signals can be generated by changing the value of *η_e_*. When *η_e_* = 92.59 mV/s, the model outputs a low-frequency signal with sporadic spikes only at the beginning. When *η_e_* = 277.78 mV/s, the model output signal has an obvious α rhythm component, and the frequency is mainly concentrated at 10 Hz. At this time, both the values of *G_e_* and *η_e_* are close to the typical physiological values. When *η_e_* = 462.96 mV/s, the simulated signal has a regular spike wave that is slightly shifted compared to the *α* rhythm. Specifically, both the amplitude peak and the normalized spectrum peak of the model’s output signal increase, and the frequency is still concentrated at 10 Hz. When *η_e_* = 648.15 mV/s, the outputted EEG signals of the model are re-transformed into an *α* waveform, the amplitude peak is further increased, and the frequency is stable at 10 Hz. Therefore, we can conclude from Figure 7 that the amplitude peak of the model’s output signal increases with *η_e_*, and there is no frequency shift.

### 3.2. The Effect of η_i_ on the Model

**Figure 8:**
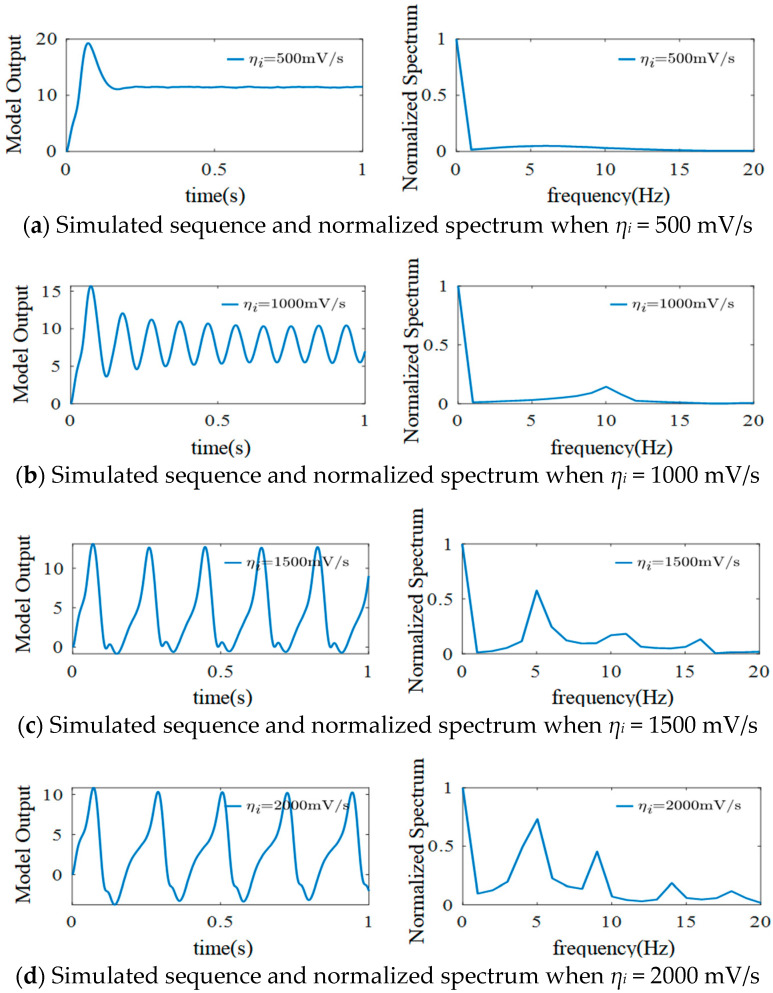
Simulated EEG signal curves and the normalized spectrum with different *η_i_*.

Similar to the discussion on the effects of *η_e_*, this section explores the effects of changing the value of inhibitory synaptic gain *η_i_* on the simulation results. Setting *η_e_* = 325 mV/s and keeping other parameters unchanged gives the EEG simulation results shown in Figure 8.

As shown in Figure 8, when *η_i_* = 500 mV/s, the output signals do not fluctuate significantly, there are sporadic spikes in the initial stage, and the normalized spectrum does not show a peak. When *η_i_* is increased from 500 mV/s to 1000 mV/s, the output EEG signals are transformed into an α waveform (see the left column of (b)), and the frequency is concentrated at 10 Hz, as shown in the right column of Figure 8b. However, α rhythms disappear, and regular spikes appear when *η_i_* is increased from 1000 mV/s to 1500 mV/s. The frequencies in the normalized spectrum are mainly concentrated in the range of 3–16 Hz, showing frequency harmonics; that is, the frequency peak appears at 5 Hz, 10 Hz, and 15 Hz and decreases in turn. When *η_i_* is increased to 2000 mV/s, the α feature of the simulated EEG signals disappears, and regular spikes appear. In the normalized spectrum, the frequencies are mainly concentrated between 3 and 20 Hz, and frequency harmonics remain. The frequency peaks appear at 5 Hz, 10 Hz, 15 Hz, and close to 20 Hz. Moreover, the spectrum peaks also decrease, but their values are more obvious. Changing the value of *η_i_* shows that, in addition to the main frequency, the simulated signal spectrum can generate frequency harmonics, which are integral multiples of the main frequency. This is similar to the frequency features of EEG signals generated by steady-state visual induction.

### 3.3. The Effects of External Inputs on the Model

#### 3.3.1. The Effect of External Inputs’ Mean Value

**Figure 9:**
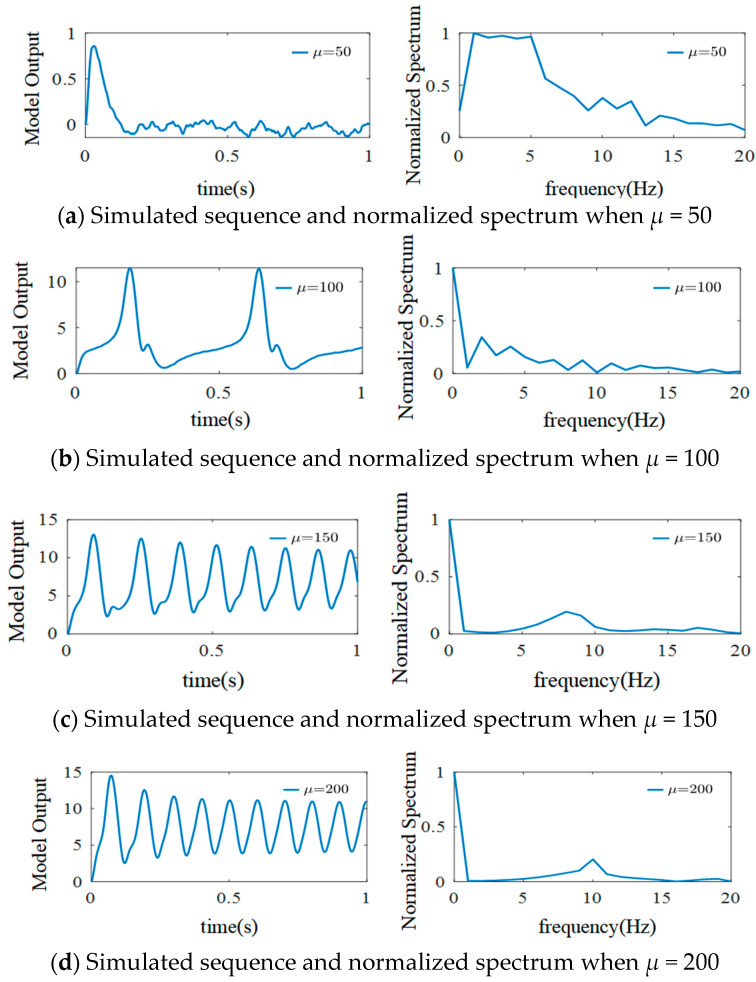
Simulated EEG signal curves and the normalized spectrum with different *μ*.

In the single-channel basic neuron mass model, all the signals in the variable regions and external stimuli are represented by Gaussian white noise *n*(*t*)~*N*(*μ*,*σ*^2^) as excitatory input. *μ* is the mean value of *n*(*t*), indicating the average strength of external input. Therefore, the effect of external input on the output signals of a simulated single-cell mass can be explored by changing the value of *μ*. Setting *σ*^2^ = 100, basic sampling time = 1/1024 s, *η*_e_ = 325 mV/s, *η_i_* = 1100 mV/s, *ω_e_* = 100 s^−1^, and *ω_i_* = 50 s^−1^ in the single-channel basic neuron mass model gives the output signals shown in Figure 9.

As shown in Figure 9, when *μ* = 50, the output signals have more low-frequency components, and sporadic spikes appear only at the beginning. When *μ* is increased from 50 to 100, regular spikes appear in the signals, and their frequencies are mainly centered in the 1–11 Hz range. Furthermore, when *μ* is increased from 50 to 150, a large number of regular spikes with a relatively obvious α waveform sign appear in the simulated signals, and there is a spectrum peak at 7–9 Hz. The rhythm characteristics are strengthened when *μ* is increased to 200, and a spectral peak appears at 10 Hz.

#### 3.3.2. The Effect of External Inputs’ Variance

**Figure 10:**
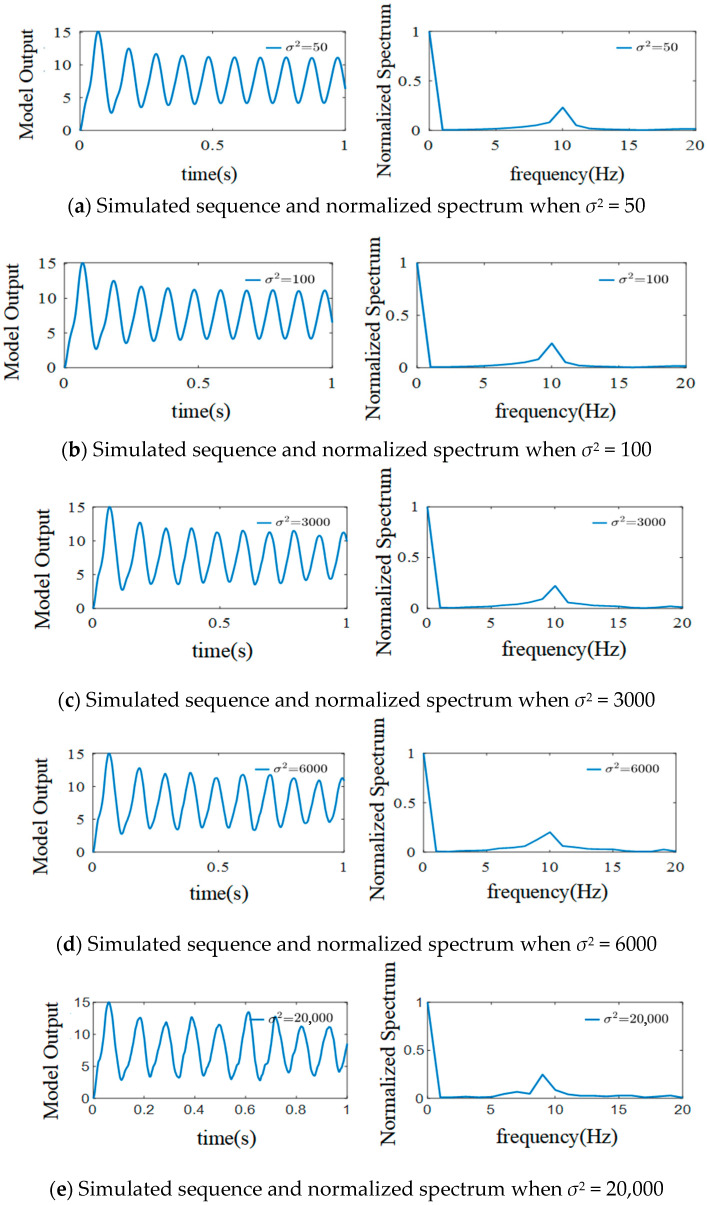
Simulated EEG signal curves and the normalized spectrum with different *σ*^2^ when *μ* = 220.

In *n*(*t*), when the average strength of the external inputs is determined, the variance *σ*^2^ can be used to represent their degree of fluctuation. The effects of *σ*^2^ on EEG signals when *μ* = 220 and the other parameters are kept constant are shown in Figure 10.

As illustrated in Figure 10, when *σ*^2^ is increased from 50 to 100, all the simulated signals of the model show *α* waveform characteristics, the frequencies are concentrated at 10 Hz, and the amplitude and spectrum do not change. Since the changes in the simulated signal are too weak, the value of *σ*^2^ is increased from 100 to 3000. In this situation, the values of the amplitude of the simulated signals change slightly, but the normalized spectrum remains unchanged. The *α* waveform characteristic of the simulated signals is still obvious, and the frequencies are still concentrated at 10 Hz when *σ*^2^ is increased from 3000 to 6000; however, the peak amplitude and frequency of the simulated signals change slightly. Further increasing *σ*^2^ to 20,000 results in the α rhythm changing significantly (although the output signals still show an α rhythm) and the frequency peak shifting to the left.

**Figure 11:**
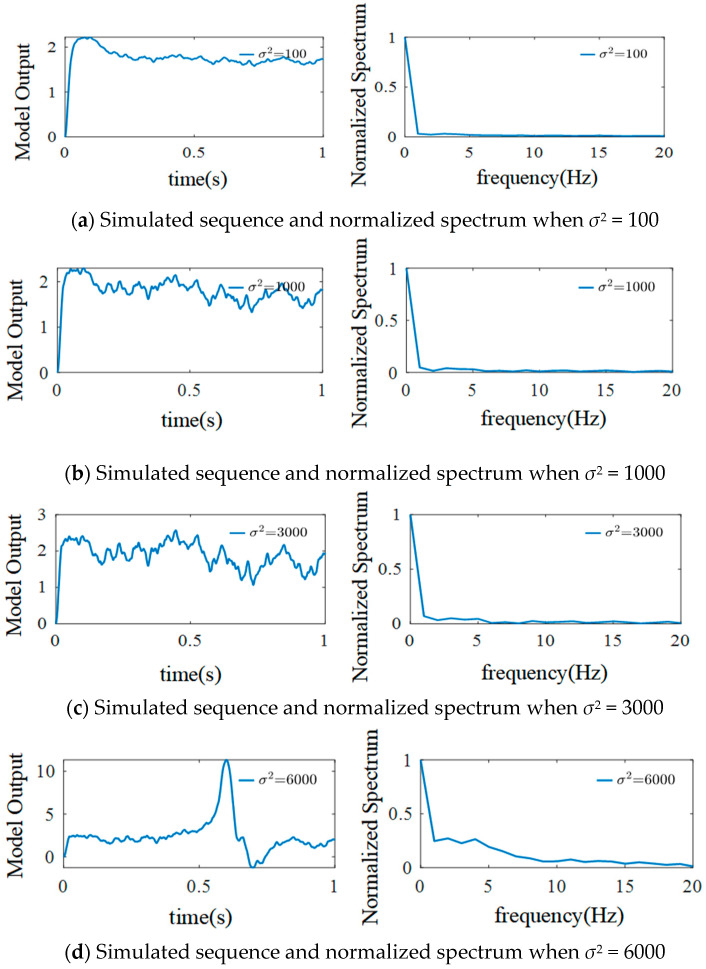

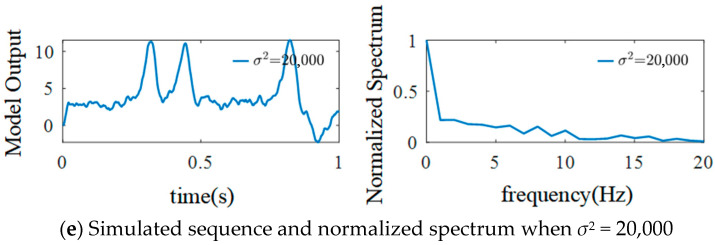
Simulated EEG signal curves and the normalized spectrum with different *σ*^2^ when *μ* = 90.

There is no obvious characteristic change after adjusting the external inputs’ fluctuation degree when the average strength of external inputs is 220, considering that when *μ* = 220, the model outputs the standard *α* rhythm. To further evaluate the effect of *σ*^2^ on the generated signals, *μ* is adjusted to 90, and the other parameters are kept consistent with those for *μ* = 220. Figure 11 shows the results.

The simulation results show that when *μ* = 90 and *σ*^2^ = 100, the simulation signals of the model exhibit weak oscillations, and there is no frequency peak. When *σ*^2^ is increased from 100 to 1000 and then from 1000 to 3000, the amplitude range of the model simulation signals’ oscillations increases significantly without frequency peaks. Sporadic spikes appear in the model simulation signals, and the spectrum changes when *σ*^2^ is increased from 3000 to 6000. Furthermore, when *σ*^2^ is increased to 20,000, a large number of spikes appear in the simulation signals, and frequency peaks appear in the spectrum, which are mainly concentrated at 5–10 Hz. In summary, when the average strength of external inputs is adjusted from 220 to 90, the simulation signals of the model change significantly with different *σ*^2^ values.

### 3.4. The Effect of Mean Time Constant on the Model

The above analyses of the effects of *η*, *μ*, and *σ*^2^ on the waveform and frequency of single-cell mass simulation signals are analyzed. Changing the average time constant *ω*^−1^ affects the value of *η* and changes other parts of the dynamic linear transformation function in Equation (4).

**Figure 12:**
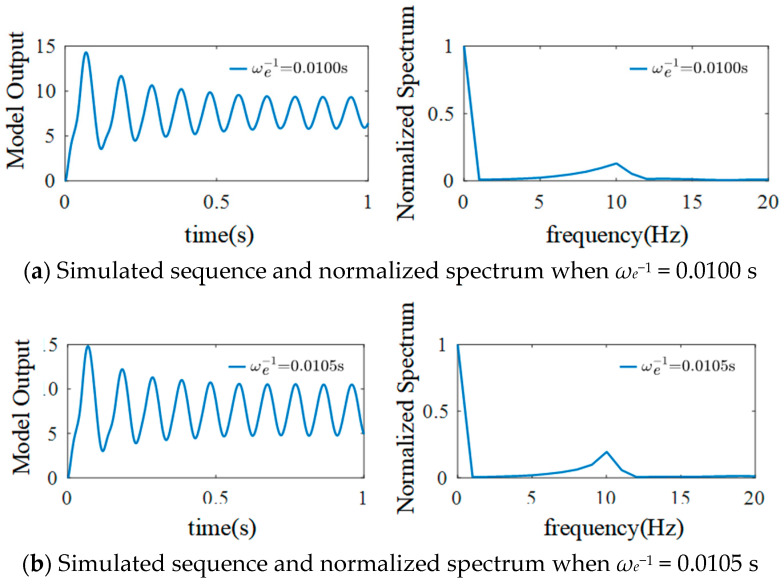

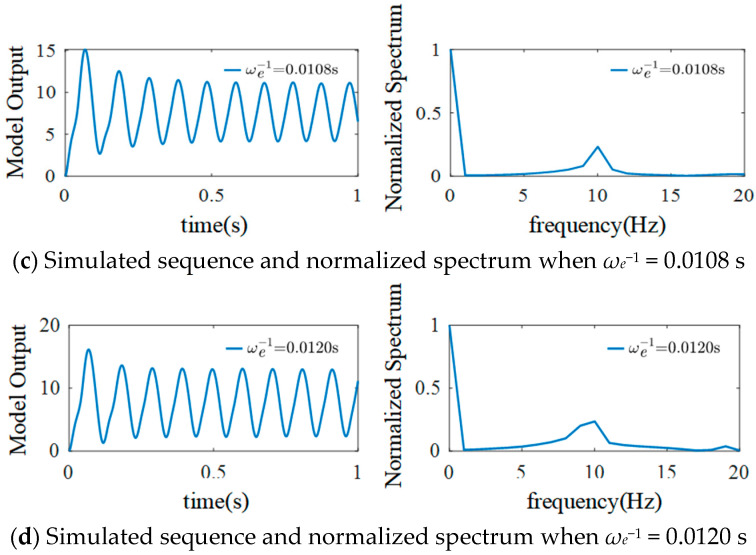
Simulated EEG signal curves and the normalized spectrum with different *ω_e_*^−1^.

When *μ* = 220, *σ*^2^ = 100, and the basic sampling time is 1/1024 s, *η_e_* represents the product of the average value of excitability synaptic gain *G_e_* and the reciprocal of the average time constant *ω_e_*. Keeping G*_e_* = 3.25 mV, *η_i_* = 1100 mV/s, and *ω_i_* = 50 s^−1^ to analyze the effect of excitability time constant *ω_e_*^−1^ on the output EEG signals of the model gives the results shown in Figure 12.

Figure 12 shows that as *ω_e_*^−1^ increases, the number of spikes of simulated EEG signals gradually decreases, with the peak values gradually increasing and moving to the left, and the spectrum peak value also gradually increasing. The excitability time constant affects the spike amplitude, spike number, spectrum peak value, and spectrum amplitude of the simulated EEG signals the model outputs.

**Figure 13:**
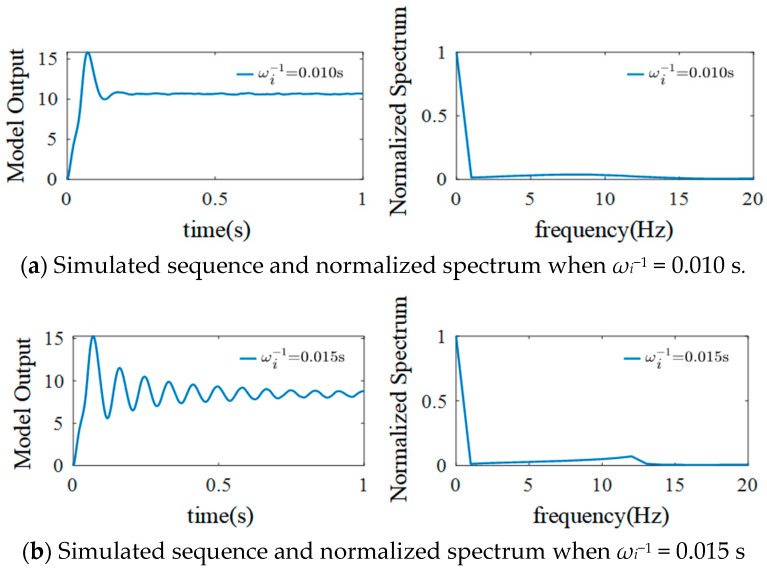

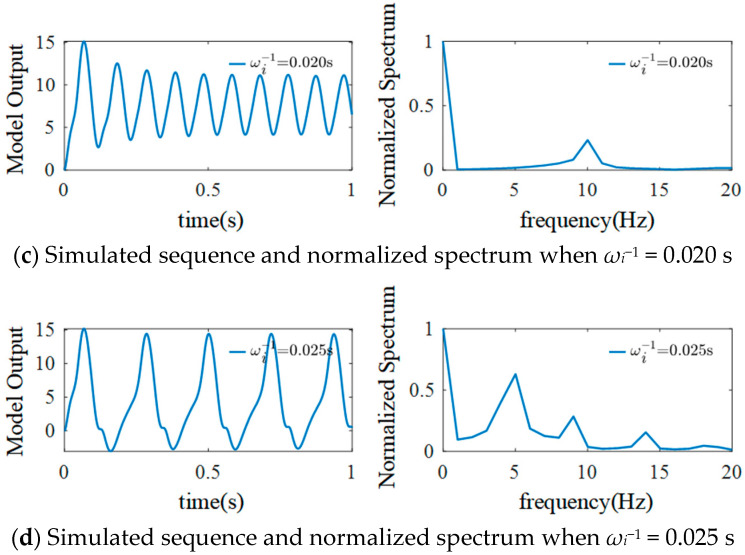
Simulated EEG signal curves and the normalized spectrum with different *ω_e_*^−1^.

In addition to the excitability time constant, the inhibitory time constant *ω_i_*^−1^ can also affect the simulated EEG signals. To explore this, the following parameters were set: *μ* = 220, *σ*^2^ = 100, basic sampling time = 1/1024 s, *G_i_* = 22 mV, *η_e_* = 325 mV/s, and *ω_e_* = 100 s^−1^. *η_i_* represents the product of the inhibitory mean synaptic gain *G_i_* and the reciprocal average time constant *ω_i_*. The simulation results are shown in Figure 13.

As shown in Figure 13, when *ω_i_*^−1^ = 0.010 s, the EEG signals outputted by the model show sporadic spikes at the beginning and then oscillate weakly. Moreover, there is no peak value in the normalized spectrum. When *ω_i_*^−1^ is increased to 0.015 s, the amplitude of EEG signals increases, and they show an α rhythm. The spike peaks gradually decrease, and the spectrum peak appears at 10–11 Hz. The amplitude of the EEG signals further increases when *ω_i_*^−1^ is increased to 0.020 s. At the same time, the number of signal spikes decreases, and the spectrum peak shifts to the left, but its value increases. When *ω_i_*^−1^ is further increased to 0.025 s, regular spikes appear in the output signals, α rhythm characteristics gradually disappear, signal amplitude increases, the number of spikes decreases, and spectrum harmonics are present. The spectrum peaks appear at 5 Hz, 10 Hz, and 15 Hz, and their values decrease successively.

## 4. Discussion and Conclusions

Based on the feedback mechanism and principle of the basic neuron mass model, this study introduced the static nonlinear information transformation process and dynamic linear information transformation process of the model, with a detailed explanation of the physiological parameters and their physical significance. Through simulation analysis of a single-channel basic NMM, we thoroughly investigated the effects of key parameters on EEG signal output characteristics and their normalized spectra.

The simulation results of the model demonstrated that the average synaptic gain *G*, the average time constant *ω*^−1^, and the external input *n*(*t*) are the primary factors affecting EEG signal output. Specifically, the mean intensity *μ* and fluctuation degree *σ*^2^ of the external input *n*(*t*) jointly influence the model’s output, with particularly pronounced effects when *σ*^2^ undergoes significant changes. Furthermore, by adjusting the inhibitory product *η_i_* and inhibitory time constant *ω_i_*⁻^1^, we observed the emergence of frequency harmonics in the spectral analysis of the simulated signals, occurring at integer multiples of the fundamental frequency. This phenomenon closely aligns with the characteristics of fundamental and harmonic responses in SSVEP signals, providing theoretical support for understanding the physiological mechanisms of SSVEP.

While this research employed a basic NMM to analyze SSVEP response mechanisms, several critical limitations must be acknowledged.

First, the current model relies on white Gaussian noise as the sole input perturbation, which oversimplifies the stochastic nature of neuronal activity. Real cortical dynamics are influenced by a mixture of white and pink noise (1/f noise), with pink noise dominating low-frequency components. This omission may restrict our ability to capture low-frequency phase coherence and stochastic resonance effects critical for SSVEP generation [35]. For instance, pink noise’s energy distribution could enhance thalamocortical entrainment of *γ*-band oscillations (30–80 Hz), amplifying SSVEP amplitude through nonlinear interactions [36]. Therefore, the absence of pink noise in our simulations may underestimate the stability and frequency specificity of SSVEP responses in realistic scenarios.

Second, although this study concentrated on narrowband behavior, its limited capability to capture multiple dynamic patterns constitutes a critical methodological constraint. While our model effectively captures SSVEP generation within local V1 circuits, it fails to account for the rich poly-frequency oscillatory dynamics observed in real brains. David et al. [37] proposed multi-dynamic neuron population models (multi-dynamic NMMs) that capture complex oscillatory dynamics in specific brain regions, such as the spatiotemporal coupling of *γ*-band rhythms and *θ-α* cross-frequency interactions, thereby establishing a more comprehensive dynamical framework for SSVEP analysis.

Third, this research focused on a single-channel basic NMM, which lacks the capacity to model interregional neural coupling mechanisms, such as functional interactions between the occipital visual cortex and frontal executive control regions. These cross-brain synchronization processes—exemplified by theta–alpha cross-frequency coupling during attentional modulation—not only directly influence SSVEP amplitude modulation but also contribute to the spatiotemporal organization of phase locking [6]. Neglecting these coupled dynamics risks fundamental misinterpretations of the hierarchical structure in SSVEP-related neural networks [38].

Currently, the development of SSVEP-based brain–computer interface (BCI) devices is constrained by experimental equipment, conditions, and individual subject variations. Acquiring real SSVEP signals involves high experimental costs, and insufficient data volume limits further research. Recent studies have attempted to generate SSVEP data consistent with real signal distributions through deep learning methods for data augmentation. However, these generation methods typically maintain mathematical distribution consistency with original data while lacking physiological interpretability and credibility, making it challenging to fully meet practical SSVEP signal modeling requirements.

Looking ahead, to address the inherent limitations of current NMMs and ensure the reliability and physiological significance of generated signals, future research should explore the development of hybrid generation frameworks that synergize systematically optimized neuron population models (NMMs) with deep learning techniques. The NMM could provide a signal generation framework based on neurophysiological mechanisms, ensuring the biological plausibility of generated signals. Meanwhile, deep learning methods could optimize generated signals using actual data distribution characteristics, enhancing the model’s ability to fit real signals. This integration would not only address SSVEP data insufficiency but also enable the real-time generation of biologically meaningful signals for practical BCI device control and optimization. This exploration direction presents new opportunities for SSVEP-based BCI technology development while promoting the convergence of neuroscience and artificial intelligence.

## Figures and Tables

**Table 1:** Physiological significance and typical values of the model parameters.

Parameters	Physiological Significance	Typical Values		
		*δ* Waveform	*α* Waveform	*γ* Waveform
*η* *_e_*	G_e *_ ω_e_	40 mV/s	325 mV/s	1630.43 mV/s
*η* *_i_*	G_i *_ ω_i_	300 mV/s	1100 mV/s	51,724.14 mV/s
*ω_e_* ^−1^	Excitability time constant	0.05 s	0.0108 s	0.0046 s
*ω_i_* ^−1^	Inhibitory time constant	0.05 s	0.02 s	0.0029 s
*C*_1_, *C*_2_	Excitatory mean synaptic connections	*C*_1_ = *C*, *C*_2_ = 0.8*C* (*C* = 135)		
*C*_3_, *C*_4_	Inhibitory mean synaptic connections	*C*_3_ = 0.25*C* *C*_4_ = 0.25*C*		
*v*_0_, *e*_0_, *r*	Parameters of the nonlinear function	*v*_0_ = 6 mV, _0_ = 2.5 s^−1^, *r* = 0.56 mV^−1^		

## Data Availability

All data generated or analyzed to support the findings of this study are included within the article.
